# Insulin Analogue Levemir and Development of Pancreatic Adenocarcinoma: A Case Report and Literature Review

**DOI:** 10.4021/jocmr936w

**Published:** 2012-07-20

**Authors:** Aditya Arya, Noori Al-Waili

**Affiliations:** a Heath and Wellness Center, Mount Vernon, New York, USA

**Keywords:** Levemir, Adenocarcinoma, Pancreas

## Abstract

There is an association between insulin analogues, glargine, lispro and aspart and the incidence of malignancies. A 76 year-old female patient with history of diabetes, hypertension and hyperlipidemia presented with flank pain after using insulin Levemir as part of her management. CT abdomen revealed pancreatic mass and endoscopic ultrasound- guided FNA aspiration biopsy showed pancreatic adenocarcinoma. It was not clear whether the insulin analogue initiated the tumor or helped the growth of pre-existing malignant foci. Then we concluded that case presented raises an indicator of possible association between using insulin analogue and initiation of malignancies, or stimulation of malignant foci.

## Introduction

A positive association between the daily insulin dosage (glargine, lispro and aspart) and the incidence of malignancies was found. After adjusting for insulin dose, a dose-dependent increased risk of cancer was found in patients treated with insulin glargine compared with patients treated with human insulin [1]. Another study showed that patients taking insulin glargine had an almost twofold increased risk of breast cancer compared with patients using other types of insulin [2]. The Scottish Diabetes Research Network Epidemiology Group similarly observed an increased risk [3]. However, no association between insulin analogues and cancer progression as compared with human insulin was found, but using any kind of insulin showed a higher risk of developing adenocarcinomas than those on metformin [4]. In a meta-analysis performed in 8,693 patients with type 1 or type 2 diabetes, who were included in Novo Nordisk-sponsored, randomized and controlled diabetes trials, it was found that patients treated with insulin detemir had a lower or similar occurrence of cancer compared with patients treated with NPH insulin or insulin glargine [5].

Here we present a care report of the development of pancreatic adenocarcinoma after the use of insulin Levemir.

## Case Report

A 76 year-old female patient presented with 1 week history of intermittent, sharp, left flank pain, rated 8/10 in intensity. She denied nausea, vomiting, diarrhea, constipation, fever, trauma, or bleeding. She reported a similar painful episode 4 weeks prior.

Her medical history is significant for a 10-year history of diabetes mellitus, hypertension, hyperlipidemia, and thyroidectomy for a thyroid nodule over 25 years ago. On physical examination, the patient was afebrile with an elevated BP of 160/80 and a pulse of 90 beats per minute. Her abdominal exam was remarkable for tenderness in left mid quadrant with deep palpation, without any appreciable organomegaly or masses. There were no pulsations or bruits, and her abdomen was soft and non-distended. Her rectal exam was remarkable for hemorrhoids and a skin tag, with no bright red blood in the vault.

The Abdominal US was unremarkable. Her laboratory investigations were within normal limits except for glucose 377 and HgbA1C: 9.6%.

Her diabetic medication regimen history included treatment with Metformin and Glyburide. Januvia was started December, 15, 2009. Thereafter Glyburide was stopped and Novolog Mix was started. Due to uncontrolled blood glucose, the regimen was changed to Lantus and Humalog on December 29, 2009 with an endocrinology follow-up. Patient had some confusion with the recently changed regimen, and when she had returned after two weeks she was requested to take Levemir 10 units daily and continue Humalog 6 units TID with meals. She was on the Levemir and Humalog combination when she was seen for abdominal pain.

After 1 week, she reported an exacerbation of the abdominal pain, which prompted her to seek care in the ER where a CT of the abdomen was done. The CT was showed a mass in the head of the pancreas, highly suggestive of a malignant neoplasm.

The patient was underwent an ERCP with cytology which was remarkable for mild dilation of the pancreatic and bile ducts at the level of the ampula with a negative cytology for malignant cells. The patient was discharged for outpatient follow-up with surgery and GI consults, where an endoscopic ultrasound guided FNA aspiration biopsy was positive for adenocarcinoma. The mass was inoperable and the patient was given consultation for oncology/chemotheraphy. The patient declined chemotherapy andwas discharged to hospice care as per her wishes.

## Discussion

The case presented herein showed that the patient developed abdominal pain 4 - 5 months after starting Levemir and was diagnosed with inoperable pancreatic adenocarcinoma eight months later. It was not clear whether the patient had pancreatic cancer started before using Levemir or developed after using Levemir. Insulin analogues were developed to improve the pharmacological properties of injected insulin, and therefore, they are widely used in the treatment of diabetes mellitus. IGF-1 is a growth factor involved in cancer initiation and progression. Insulin analogs such as glargine, detemir and lispro have had proliferative effects that resemble IGF-I action [6]. It was concluded that insulin glargine and insulin detemir display atypical signaling activities that differ from those elicited by regular insulin and involve activation of the anti-apoptotic IGF- insulin receptor [7].

Recently, analysis of signaling pathways in human mammary epithelial cell lines stimulated by insulin analogs revealed that regular insulin and glargine strongly activate the IGF- insulin receptor and the MAP kinase pathway in MCF7 cells; glargine is a strong mitogen for cells characterized by a high-IGF- insulin receptor/insulin receptor ratio [8]. In patients with type-1 diabetes, serum containing insulin glargine was 1.11 fold more mitogenic than human insulin-containing serum; mitogenicity of serum containing detemir was 0.99 fold that of human insulin-containing serum [9].

In vitro experiments revealed that all kinds of insulin compounds including the long-acting insulin analogue glargine enhance capillary-like tube formation [6]. Insulin receptors were found to be strongly expressed on the endothelium of microvessels in human adenocarcinoma (breast, colon, pancreas, lung and kidney). Incubation with commercially available insulin compounds increased capillary-like tube formation of human microvascular endothelial cells in vitro [10].Distinctive insulin compounds could contribute to tumor growth by enhancing local angiogenesis [8].

Generally, there is no enough evidence to support that insulin does indeed influence cancer incidence by induction of malignancies, or it has stimulatory effects on pre-existing malignant foci. The case presented herein raises an indicator of possible association between using insulin analogue and initiation of malignancies, or stimulation of malignant foci. It should be emphasized that further prospective controlled trials will be required to confirm a possible relationship between insulin therapy and tumor development and progression.
